# IgA regulates the composition and metabolic function of gut microbiota by promoting symbiosis between bacteria

**DOI:** 10.1084/jem.20180427

**Published:** 2018-08-06

**Authors:** Akira Nakajima, Alexis Vogelzang, Mikako Maruya, Michio Miyajima, Megumi Murata, Aoi Son, Tomomi Kuwahara, Tatsuaki Tsuruyama, Satoshi Yamada, Minoru Matsuura, Hiroshi Nakase, Daniel A. Peterson, Sidonia Fagarasan, Keiichiro Suzuki

**Affiliations:** 1 Center for Innovation in Immunoregulative Technology and Therapeutics, Graduate School of Medicine, Kyoto University, Kyoto, Japan; 2 Laboratory for Mucosal Immunity, Center for Integrative Medical Sciences, RIKEN Yokohama Institute, Yokohama, Japan; 3 Department of Microbiology, Faculty of Medicine, Kagawa University, Kagawa, Japan; 4 Center for Anatomical, Pathological, Forensic Medical Research and Department of Drug and Discovery Medicine, Graduate School of Medicine, Kyoto University, Kyoto, Japan; 5 Department of Gastroenterology and Hepatology, Graduate School of Medicine, Kyoto University, Kyoto, Japan; 6 Department of Gastroenterology and Hepatology, School of Medicine, Sapporo Medical University, Sapporo, Japan; 7 Department of Pathology, Johns Hopkins University School of Medicine, Baltimore, MD

## Abstract

Immunoglobulin A (IgA) promotes health by regulating the composition and function of gut microbiota, but the molecular requirements for such homeostatic IgA function remain unknown. We found that a heavily glycosylated monoclonal IgA recognizing ovalbumin coats *Bacteroides thetaiotaomicron* (*B. theta*), a prominent gut symbiont of the phylum Bacteroidetes. In vivo, IgA alters the expression of polysaccharide utilization loci (PUL), including a functionally uncharacterized molecular family provisionally named Mucus-Associated Functional Factor (MAFF). In both mice and humans, MAFF is detected predominantly in mucus-resident bacteria, and its expression requires the presence of complex microbiota. Expression of the MAFF system facilitates symbiosis with other members of the phylum Firmicutes and promotes protection from a chemically induced model of colitis. Our data reveal a novel mechanism by which IgA promotes symbiosis and colonic homeostasis.

## Abstract (precis)

IgA regulates the composition and function of gut microbiota. Nakajima et al. show that a heavily glycosylated monoclonal IgA coats *B. theta* and induces Mucus-Associated Functional Factor in vivo to enhance symbiotic interactions with commensal bacteria to maintain gut homeostasis.

## Graphical Abstract

**Figure:**
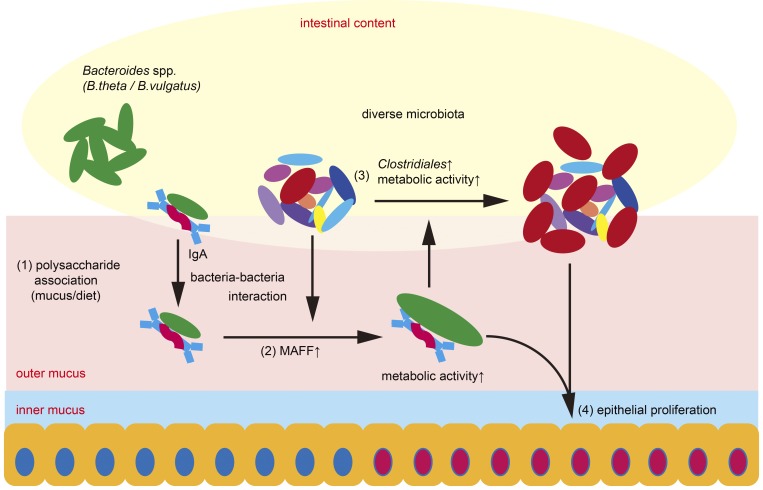

## Introduction

The gut microbiota is essential for host physiology, as it regulates the metabolism, epithelial barrier integrity, and immune system development and function (Bäckhed et al., 2005; Artis, 2008; Belkaid and Hand, 2014). Numerous studies have revealed that dietary, environmental, and host-derived factors have a strong impact on the composition and activity of this important microbial organ (De Filippo et al., 2010; Maurice et al., 2013; Goodrich et al., 2014). In our previous studies using immunodeficient mouse strains, we found that IgA plays an important role in controlling the composition and geographical distribution of bacterial communities along the gastrointestinal tract (Fagarasan et al., 2002; Suzuki et al., 2004; Kawamoto et al., 2012). This homeostatic role of IgA in regulating commensal bacteria was recently confirmed in humans with IgA deficiency (Fadlallah et al., 2018). In fact, a significant fraction of commensal bacteria was shown to be coated by IgA and stably maintained in homeostatic conditions (van der Waaij et al., 1996). More recently, we showed that diversification and selection of IgA repertoires in a T cell–dependent manner in germinal centers of the Peyer’s patches contribute to enhancing the diversity and stability of gut-resident species (Kawamoto et al., 2014). In addition, T-independent pathways, likely originating in the lamina propria, have been shown to contribute to the generation of an IgA repertoire that is polyreactive to a broad swathe of bacteria residing in the small intestine (Bunker et al., 2015, 2017). Some of this polyreactivity, with respect to gram-positive species in particular, has been attributed to Fab fragment–independent interactions with the glycans associated with both antibody chains and the secretory component (Mathias and Corthésy, 2011).

The glycan-rich gut mucus layer forms a fundamental niche for symbionts, nurturing host–bacterial relationships by providing nutrients and a scaffold for growth (Bäckhed et al., 2005; Martens et al., 2008; Li et al., 2015). An in silico simulation predicted that bacterial adhesion to host factors, especially that of mucus glycans and IgA, would enhance the bacterial competition and contribute to the selection of microbial community in the gut (McLoughlin et al., 2016). As IgA and bacteria are both abundant within the mucus layer, particularly the outer mucus of the colon, IgA may enhance commensal colonization of this microbial niche by promoting adhesion and/or nutrient utilization of bacteria within the colonic mucus (Johansson et al., 2008; Rogier et al., 2014).

We tested whether “bystander” IgA (that is, IgA elicited by antigens other than bacterial antigens) can modulate gut microbiota via glycan–glycan interactions among IgA, bacteria, and mucus. To do this, we generated a monoclonal IgA (7-6IgA) recognizing OVA, which is heavily glycosylated. We show that 7-6IgA efficiently binds to the human symbiont *Bacteroides thetaiotaomicron* (*B. theta*) and modulates the gene expression profile of this bacterium in vivo. We find that a subset of mucus-resident *B. theta* specifically up-regulated a set of functionally uncharacterized genes in response to IgA and diverse microbiota in mouse models, as well as in samples from healthy human colon. Mechanistic analyses revealed that these *B. theta* molecules are essential during competitive colonization and provide protection against chemically induced colitis, leading to expansion and altered metabolic activity of bacteria in the phylum Firmicutes, particularly order Clostridiales. This study shows that bystander IgA has the capacity to modulate gene expression and function of members of the gut microbial community, promoting symbiosis between commensal bacterial species required for colonic homeostasis.

## Results

### Heavily glycosylated monoclonal IgA recognizing OVA efficiently coats *B. theta*

**Figure 1.**
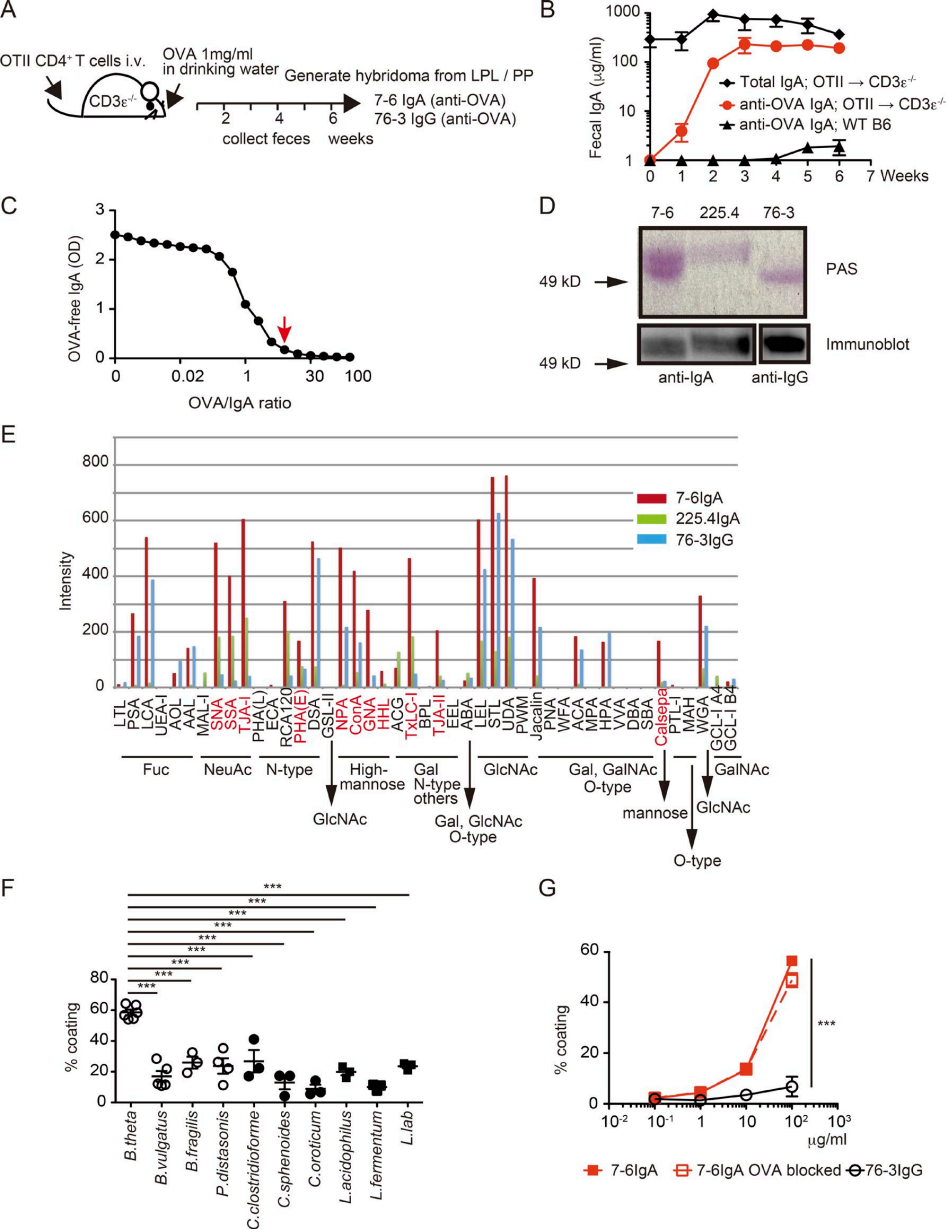
**Generation and characterization of 7-6IgA. (A)** Experimental scheme for the generation of hybridoma cells. Briefly, OTII-Tg CD4^+^ T cells were transferred into CD3ε^−/−^ mice given 1 mg/ml OVA in drinking water. Hybridoma cells were prepared with mucosal lymphocytes 6 wk after transfer. LPL, lamina propria lymphocytes; PP, Peyer’s patches. **(B)** ELISA measurement of total and anti-OVA IgA in fecal samples collected from CD3ε^−/−^ mice transferred with OTII-Tg CD4^+^ T cells, as in A, and control C57BL6 WT type mice, which received oral OVA. *n* = 3–19 mice for each point. **(C)** Competitive antigen-binding assay to measure OVA binding capacity. Purified 7-6IgA was preincubated with varying concentrations of OVA (OVA/IgA ratio in wt/vol is plotted on x axis), and the remaining OVA-free 7-6IgA was measured by ELISA. Arrow indicates the concentration of OVA sufficient to mask 7-6IgA antigen binding. Each dot represents one assay well, and the representative data of two similar experiments are shown. **(D)** Glycosylation of monoclonal 7-6IgA and 76-3IgG generated in A and 225.4IgA (*B. theta*–specific IgA) measured by periodic acid-Schiff (PAS) staining, and immunoblots showing equal antibody loading. The 7-6IgA data are representative of two similar experiments. **(E)** Normalized fluorescent signals from lectin microarray analysis of purified 7-6IgA, 225.4IgA, and 76-3IgG. The lectins for which the ratio of signal values both for 7-6IgA/76-3IgG > 2 and 7-6IgA/225.4IgA > 2 are shown in red, indicating glycans that may mediate 7-6IgA–specific bacterial coating. Fuc, fucose; NeuAc, N-acetylneuraminic acid (sialic acid); GlcNAc: N-acetylglucosamine; Gal, galactose; GalNAc, N-acetylgalactosamine. The data are representative of seven different concentrations of the antibodies measured under four different gain conditions. **(F)** The indicated cultured bacterial strains were incubated with 250 μg/ml of 7-6IgA, and the proportion of 7-6IgA–coated bacteria was measured by flow cytometry. *n* = 3–6 independent experiments for each group. **(G)** Flow cytometry measurements of *B. theta* coating with 7-6IgA, with and without preabsorption to OVA. Pooled data from three independent experiments. Statistical analyses were performed with one-way ANOVA with Tukey’s multiple comparison test (F) or with Student’s *t* test (G; 7-6IgA versus 76-3IgG at the highest concentration). ***P < 0.001. Error bars represent SEM.

**Figure 2.**
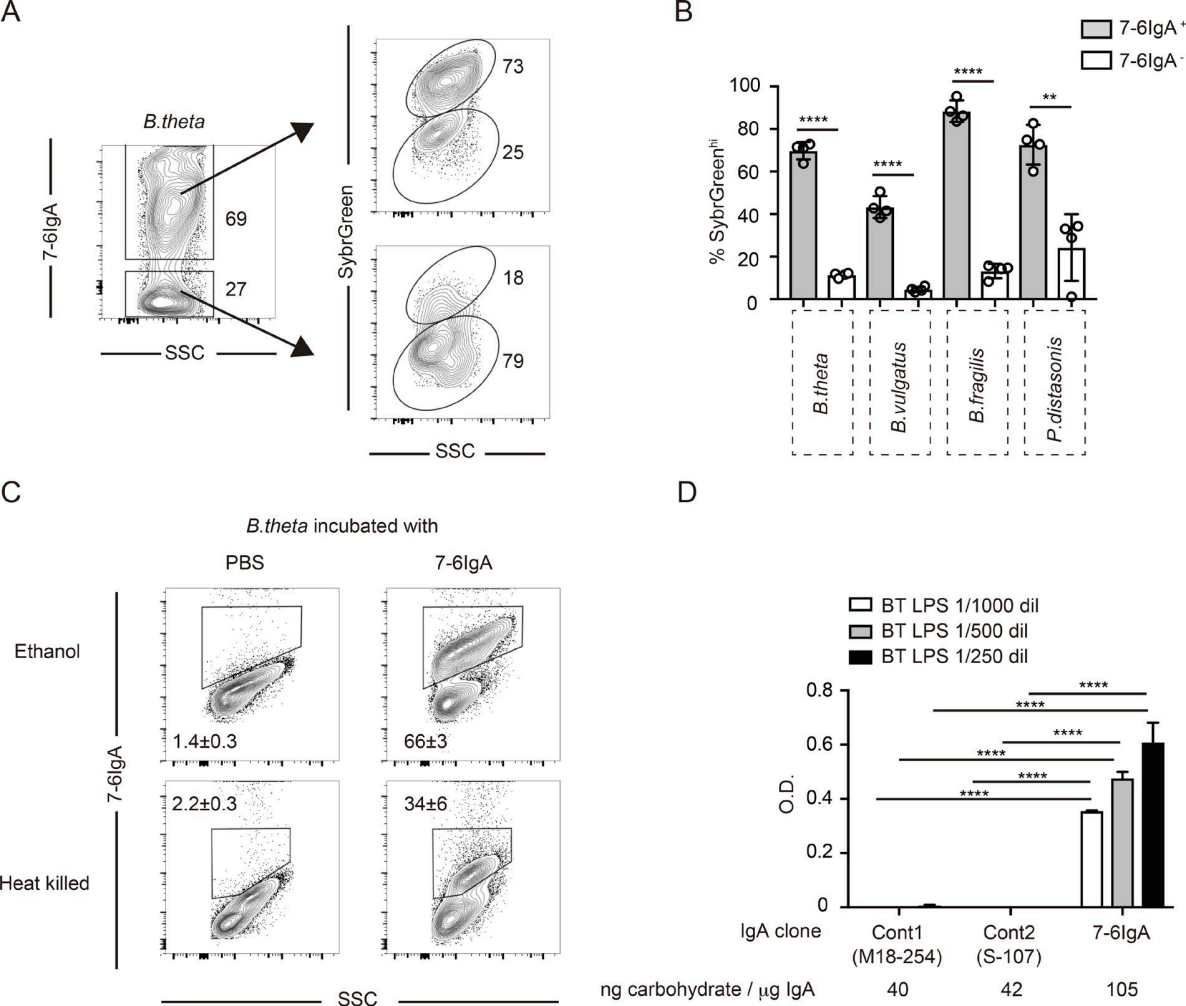
**7-6IgA binds LPS on metabolically active bacteria. (A and B)** Representative flow cytometry of cultured *B. theta* incubated with 7-6IgA and stained with anti-IgA (A) and quantification of the percent of metabolically active bacteria with high nucleic acid content (SybrGreen^hi^) of the indicated cultured Bacteroidales within both the 7-6IgA^–/+^ populations (B). Data are cumulative from four independent experiments per group. **(C)** Representative flow cytometry plots showing 7-6IgA binding to cultured *B. theta* treated with 70% ethanol (upper panels) or heat killed (70°C, 30 min; lower panels). Numbers in plots represent the mean frequency of gated cells, ±SEM of the gated fraction pooled from four independent experiments. **(D)** ELISA OD measurements of the capacity of the indicated monoclonal IgA clones to bind *B. theta*–derived LPS. Representative plot showing the mean of triplicate measurements from two similar experiments. The total amounts of carbohydrate indicated under each purified IgA clone was measured by total carbohydrate colorimetric assay. Statistical analyses were performed with unpaired Student’s *t* test (B) and with two-way ANOVA with Tukey’s multiple comparison test (D). **P < 0.01 and ****P < 0.0001. Error bars represent SEM.

We generated a system in which gut IgA was enriched in antibodies targeting the nonbacterial model antigen OVA in order to test whether bystander IgA binds and modulates commensal bacteria. Transgenic (Tg) OTII CD4^+^ T cells specific for OVA were transferred into T cell–deficient CD3ε^−/−^ mice (OTII→CD3ε^−/−^ mice), and their drinking water was supplemented with OVA to provoke a strong immune response in the gut (Fig. 1 A). 3 wk after Tg T cell transfer, the majority of fecal IgA was specific for the OVA antigen (Fig. 1 B). Flow cytometry and histological analysis confirmed that fecal and cecal bacteria from OTII→CD3ε^−/−^ mice were coated by both OVA and IgA, whereas those from control WT mice receiving oral OVA were coated in IgA alone (Fig. S1, A and B). These observations suggest that IgA–OVA immune complexes bind to bacterial surfaces even when antibody-binding sites are occupied by their cognate antigen in vivo. We next generated hybridomas from small intestine lamina propria cells of OTII→CD3ε^−/−^ mice and selected OVA-specific clones secreting polymeric IgA or monomeric IgG (clones 7-6IgA and 76-3IgG; Fig. 1 C and Fig. S1 C). Monoclonal 7-6IgA was heavily glycosylated compared with 76-3IgG, or a monoclonal IgA that has been previously described to recognize the cell wall antigen of *B. theta* (clone 225.4IgA; Peterson et al., 2007; Fig. 1 D). Assessment of the glycosylation pattern of 7-6IgA revealed abundant fucose, N-acetylneuraminic acid, high-mannose, and N-acetylglucosamine moieties, resembling the profile of human IgA in previous reports (Royle et al., 2003; Huang et al., 2015; Fig. 1 E). The highly glycosylated 7-6IgA coated all bacterial strains tested in vitro, most prominently *B. theta* (Fig. 1 F). A similar binding profile was observed with fecal polyclonal IgA obtained from OTII→CD3ε^−/−^ mice (Fig. S1 D). The coating with 7-6IgA was independent of antigen recognition, as it was unchanged by preabsorption to OVA (Fig. 1 G). We measured antibody binding to cultured *B. theta* and other members of Bacteroidales and found that 7-6IgA binding was predominant within the metabolically active SybrGreen-positive fraction (Maurice et al., 2013; Fig. 2, A and B). 7-6IgA also bound to ethanol-fixed and heat-killed *B. theta*, which suggests that 7-6IgA may bind to cell wall components generated by bacteria that are also present following bacterial death (Fig. 2 C). We considered whether 7-6IgA glycans might mediate binding to LPS on gram-negative bacteria, as shown in a previous study showing secretory IgA N-glycan interactions with gram-positive bacterial peptidoglycans (Mathias and Corthésy, 2011). Indeed, 7-6IgA, but not less glycosylated monoclonal IgA clones with lower carbohydrate content, strongly bound to LPS purified from *B. theta* (Fig. 2 D). Together, these results support the notion that glycan–LPS interactions contribute to IgA coating of bacteria independent of antibody-epitope recognition.

### 7-6IgA alters the polysaccharide utilization profile of *B. theta*

**Figure 3.**
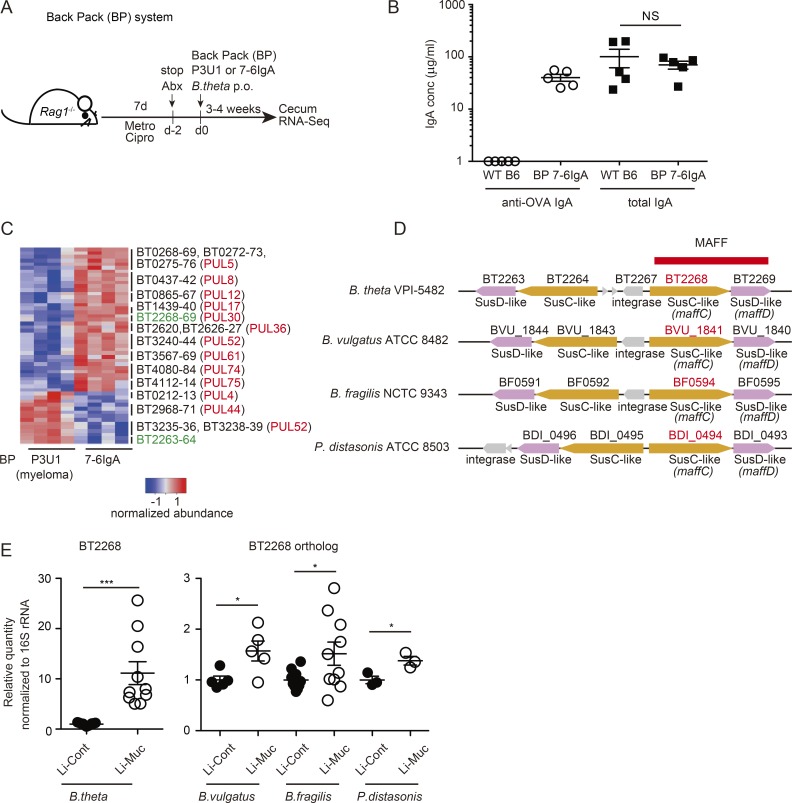
**Non-specific 7-6IgA alters gene expression profile of *B. theta* in vivo. (A)** Experimental scheme. *Rag1*^−/−^ mice were treated with metronidazole and ciprofloxacin and colonized with chloramphenicol-resistant *B. theta* upon concomitant transplantation of 7-6IgA–secreting hybridoma cells, or control parental P3U1 myeloma cells under the dorsal skin. BP, backpack. **(B)** ELISA measurements of the total and anti-OVA IgA in fecal samples collected from BP 7-6IgA mice, as in A, and age-matched C57BL6 WT mice (*n* = 5 mice for each group). **(C)** The z-score of *B. theta* PUL genes from RNA-Seq of the colonic content of BP P3U1 and BP 7-6IgA mice (*n* = 4 mice for each group). A full set of log2 FoldChange≥1 and padj≤0.05 genes is shown in Table S2. The values of variance-stabilizing transformations were used to generate the heat map. **(D)** Schematic diagram of the MAFF genes and flanking regions in the indicated Bacteroidales strains. **(E)** The expressions of BT2268 and its orthologues were measured by qPCR in indicated Bacteroidales strains. Large intestinal content (Li-Cont) and mucus (Li-Muc) were collected 4 wk after colonization of SPF WT mice previously treated with antibiotics (SPF-Abx mice; *n* = 3–11 mice). The relative values of Li-Muc in comparison with Li-Cont are plotted in the graph. Each plot in B and E and each column in C represents the result obtained from independent mice. Statistical analyses were performed with Student’s *t* test in B and E. *P < 0.05 and ***P < 0.001. Error bars represent SEM.

To test whether monoclonal 7-6IgA alters *B. theta* function in vivo, we performed *B. theta* colonization of “backpack” *Rag1^−/−^* mice, as previously described (Peterson et al., 2007; Fig. 3 A). In this system, 7-6IgA was secreted into the gut lumen at a level comparable to polyclonal IgA in WT animals (Fig. 3 B). As a control, we generated backpack mice with parental P3U1 myeloma cells, carrying a similar tumor burden without production of IgA. We then performed RNA sequencing (RNA-Seq) analysis of *B. theta* present in the cecum of backpack mice with or without 7-6IgA. This revealed that the presence of 7-6IgA significantly altered the expression levels of many genes, including polysaccharide utilization loci (PUL) transcripts in vivo (Martens et al., 2008; Koropatkin et al., 2012; Fig. 3 C and Tables S1 and S2). To identify genes that might participate in bacterial responses to IgA, we focused on the PULs that were expressed significantly only in the presence of 7-6IgA. For example, the expression of BT2268 and the nearby BT2269 was considerably up-regulated in vivo following *B. theta* colonization of 7-6IgA backpack mice (Fig. 3 C). BT2268 is a homologue of *SusC*, a TonB-dependent transporter of the starch utilization system (Sus) encoded within PUL (Martens et al., 2008). An in silico Basic Local Alignment Search Tool (BLAST) algorithm homology search revealed that there are orthologues of BT2268 in several other bacteria belonging to Bacteroidales (Fig. S2). In all these bacteria, BT2268 homologues are paired with *SusD*-homologue genes (e.g., BT2269) flanked by another *SusC/D* element in the reverse orientation (e.g., BT2264/BT2263; Glenwright et al., 2017; Fig. 3 D). We next wanted to confirm the dynamics of gene expression of BT2268 and its homologues in commensal Bacteroidales in immune replete animals with polyclonal IgA secretion. For this, we designed a system in which endogenous Bacteroidales were specifically depleted by antibiotics (specific pathogen–free [SPF] Abx mice), followed by gavage with defined Bacteroidales culture strains. Strikingly, the expressions of *B. theta* BT2268 and orthologues in *Bacteroides vulgatus*, *Bacteroides fragilis*, and *Parabacteroides distasonis* were significantly up-regulated in the mucus-resident bacteria compared with bacteria present in the colonic content (Fig. 3 E). These results indicate that 7-6IgA binding changed the polysaccharide utilization activity of *B. theta* in vivo*,* and that the BT2268/BT2269 family of molecules may act as symbiotic factors in the colonic mucus environment. We provisionally named these genes the Mucus-Associated Functional Factors (MAFFs).

### MAFF confers a competitive advantage to *B. theta*

To uncover the functional role of MAFF, we next generated *B. theta* deletion mutants for BTΔ*maffC* (ΔBT2268), BTΔ*maffD* (ΔBT2269), and BTΔMAFF (ΔBT2268/2269). These mutants were tested for their fitness both in vitro and in vivo*.* The BTΔMAFF strain grew normally in a nutrient-rich medium but showed a delayed entry into the log phase of growth in minimal medium supplemented only with glucose (MM-G; Fig. S3 A). The reduced fitness of BTΔMAFF in MM-G measured by culture density depended mostly on the function of *maffC* but not *maffD* (Fig. S3 B). We tested whether *maffC* might possess transporter activity as predicted by *maffC* homology analysis, but removing each chemical component from MM-G has not identified *maffC*-targeted molecules so far (data not shown). We next asked whether 7-6IgA binding depended on MAFF, but found that within the SybrGreen-positive fraction, the IgA coating was similar between BTΔMAFF and *B. theta* WT (BTWT) in MM-G cultures (Fig. S3 C), indicating that MAFF proteins themselves are not the target of IgA binding. Moreover, MAFF is not involved in utilization of 7-6IgA glycans, as we observed that 7-6IgA as a sole carbon source equally supported the growth of BTWT and BTΔMAFF strains in vitro (Fig. S3 D). Thus, MAFF is involved in the regulation of in vitro bacterial growth in nutrient-restricted conditions independent of 7-6IgA–glycan binding or utilization.

**Figure 4.**
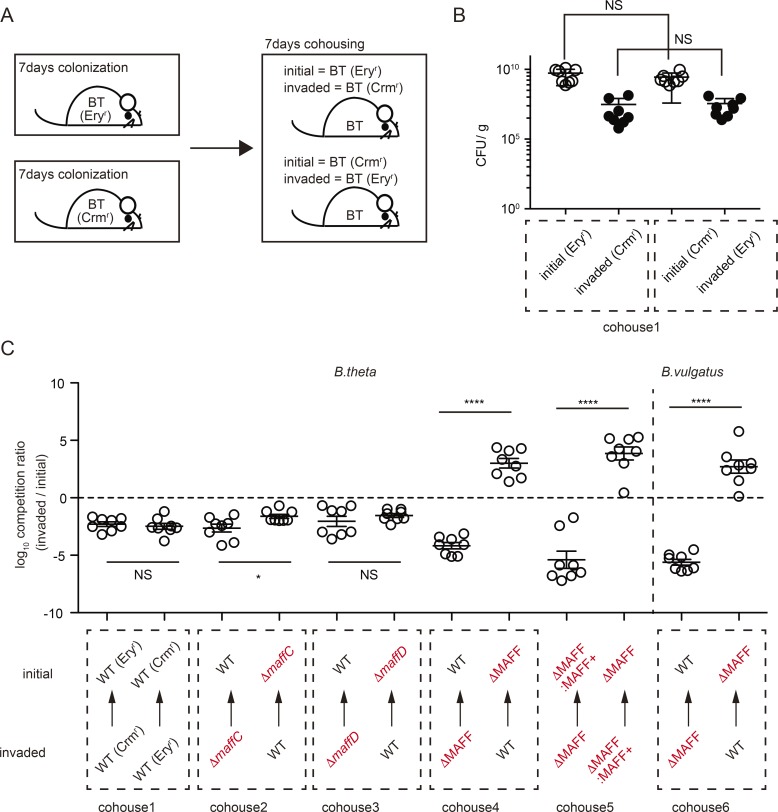
**In vivo fitness of *B. theta* and *B. vulgatus* is regulated by MAFF. (A)** Experimental scheme for cohousing of SPF-Abx mice inoculated with *B. theta* strains after antibiotic treatment. Ery^r^, erythromycin resistant; Crm^r^, chloramphenicol resistant. **(B)** CFU measurements of each *B. theta* strain in cohouse1 in C. The squares with dotted lines indicate the bacteria detected from the same mice. Each plot represents the result obtained from independent animals (*n* = 8 mice for each group). **(C)** The log_10_ ratio of invaded/initial strains in feces was determined by selective plating on day 7 of cohousing (*n* = 8 mice). The squares with dotted lines indicate the samples collected from the same group of the cohousing experiments. Statistical analyses in B and C were performed with unpaired Student’s *t* test. *P < 0.05 and ****P < 0.0001. Error bars represent SEM.

We next investigated the in vivo requirement for MAFF genes using our SPF-Abx system and performed colonization experiments with BTWT, BTΔMAFF, BTΔ*maffC*, and BTΔ*maffD*. In noncompetitive single colonization assays, all WT and mutant strains grew equally in both mucus and luminal content (Fig. S3, E and F). We then analyzed in vivo competitive fitness of BTWT and mutant strains carrying antibiotic resistance genes (Fig. 4 A), which we confirmed had no effect on colonization efficiency (Fig. 4, B and C, cohouse1). Single BTΔ*maffC* showed a mild competitive disadvantage, while BTΔ*maffD* had no colonization defect (Fig. 4 C, cohouse2 and 3). In contrast, BTWT strongly outcompeted the BTΔMAFF strain under cohousing conditions, suggesting that *maffC* and *maffD* act synergistically in vivo (Fig. 4 C, cohouse4). Indeed, complementation of the BTΔMAFF strain with *maffC/D* genes (BTΔMAFF:MAFF^+^) restored competitive persistence (Fig. 4 C, cohouse5). This effect was conserved in *B. vulgatus*, where the deletion of both MAFF genes (BVΔMAFF) also conferred reduced fitness both in vitro (Fig. S3 C) and in vivo (Fig. 4 C, cohouse6).

### MAFF functions in the context of diverse microbiota within the colonic mucus

**Figure 5.**
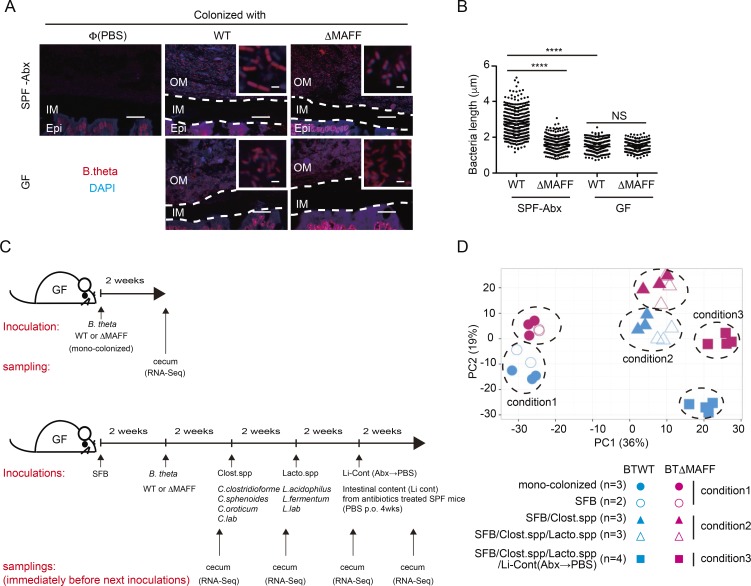
**Microbiota conventionalization influences MAFF-dependent *B. theta* phenotype in the colon. (A)** Representative confocal images showing FISH probes targeting Bacteroidales (red) and DAPI (blue) in colon sections. Antibiotics-treated (SPF-Abx) or GF mice (monocolonized) were orally inoculated with PBS or colonized with the indicated *B. theta* strains. Scale bars represent 20 µm or 2 µm on inset images. OM, outer mucus; IM, inner mucus; Epi, epithelium. **(B)** Quantification of bacterial length in FISH images, as in A. Data are (A) representative or (B) pooled from three monocolonized or six SPF-Abx mice. Each point represents a single bacteria (*n* = 353–396); 7–19 images were analyzed from each animal. Statistical analysis was performed with a Kruskal-Wallis test with Dunn’s multiple comparison test. ****P < 0.0001. **(C)** Experimental scheme. To generate monocolonized animals (upper scheme), GF mice were orally inoculated with either BTWT or BTΔMAFF. For cocolonization (lower scheme), GF mice were orally inoculated with SFB 2 wk before BTWT or BTΔMAFF colonization, and mixtures of culturable strains of *Clostridium* species (Clost.spp: mixture of *C. clostridioforme*, *C. sphenoides*, *C. oroticum*, and a laboratory-isolated strain *C. laboratory*), *Lactobacillus* species (Lacto.spp: mixture of *L. acidophilus*, *L. fermentum*, and a laboratory-isolated strain *L. laboratory*), and the intestinal contents of SPF mice 4 wk after recovery from antibiotic treatment (Li-Cont (Abx→PBS)) were added to some groups at 2-wk intervals. **(D)** Principal component analysis of BTWT and BTΔMAFF RNA-Seq transcripts from cecal content from individual gnotobiotic mice mono- or cocolonized with increasing microbiota diversity, as depicted in C. Dashed circles indicate BTWT and BTΔMAFF clusters separated on the first two principal components and paired by colonization condition. Each dot represents an individual animal (*n* = 2–4 for each group).

To gain further insight into the in vivo function of the MAFF system, we performed fluorescence in situ hybridization (FISH) with probe sets that specifically recognize *B. theta* in our SPF-Abx experimental system. Both BTWT and BTΔMAFF strains were localized in the outer mucus, but not within the inner layer or colonic crypts (Fig. 5 A). We observed that BTΔMAFF bacteria in SPF-Abx mice were significantly smaller in size when compared with their BTWT counterparts, confirming the fitness disadvantage of MAFF mutant strains in their physiological niche (Fig. 5 A, upper panels, and B). Unexpectedly, however, both BTWT and BTΔMAFF exhibited a smaller morphology in a monocolonization assay using germ-free (GF) mice (Fig. 5 A, lower panels, and B). This observation clearly indicates that *B. theta* interactions with other bacterial species not present in the GF mice are required for MAFF function.

To identify symbiotic partners, we sequentially colonized GF mice with additional, defined microbial populations and then analyzed the gene expression profile of *B. theta* using RNA-Seq (Fig. 5 C). Principal component analyses revealed marginal differences in RNA-Seq profiles of BTWT and BTΔMAFF derived from monocolonized animals, or from BTWT and BTΔMAFF mice additionally colonized with segmented filamentous bacteria (SFB), which induced a strong IgA response, as reported previously (Talham et al., 1999; Lécuyer et al., 2014; Fig. 5 D, condition1; and Fig. S3 G). Further enrichment of the colonic microbiota by addition of a small number of defined *Clostridium* spp. and *Lactobacillus* spp. strains drove a shift in the *B. theta* transcriptome compared with monocolonization, yet this was apparently independent of MAFF (Fig. 5 D, condition2). Clear separation of BTWT and BTΔMAFF RNA-Seq profiles was only observed upon the introduction of complex colonic microbiota derived from SPF-Abx mice (Fig. 5 D, condition3).

**Figure 6.**
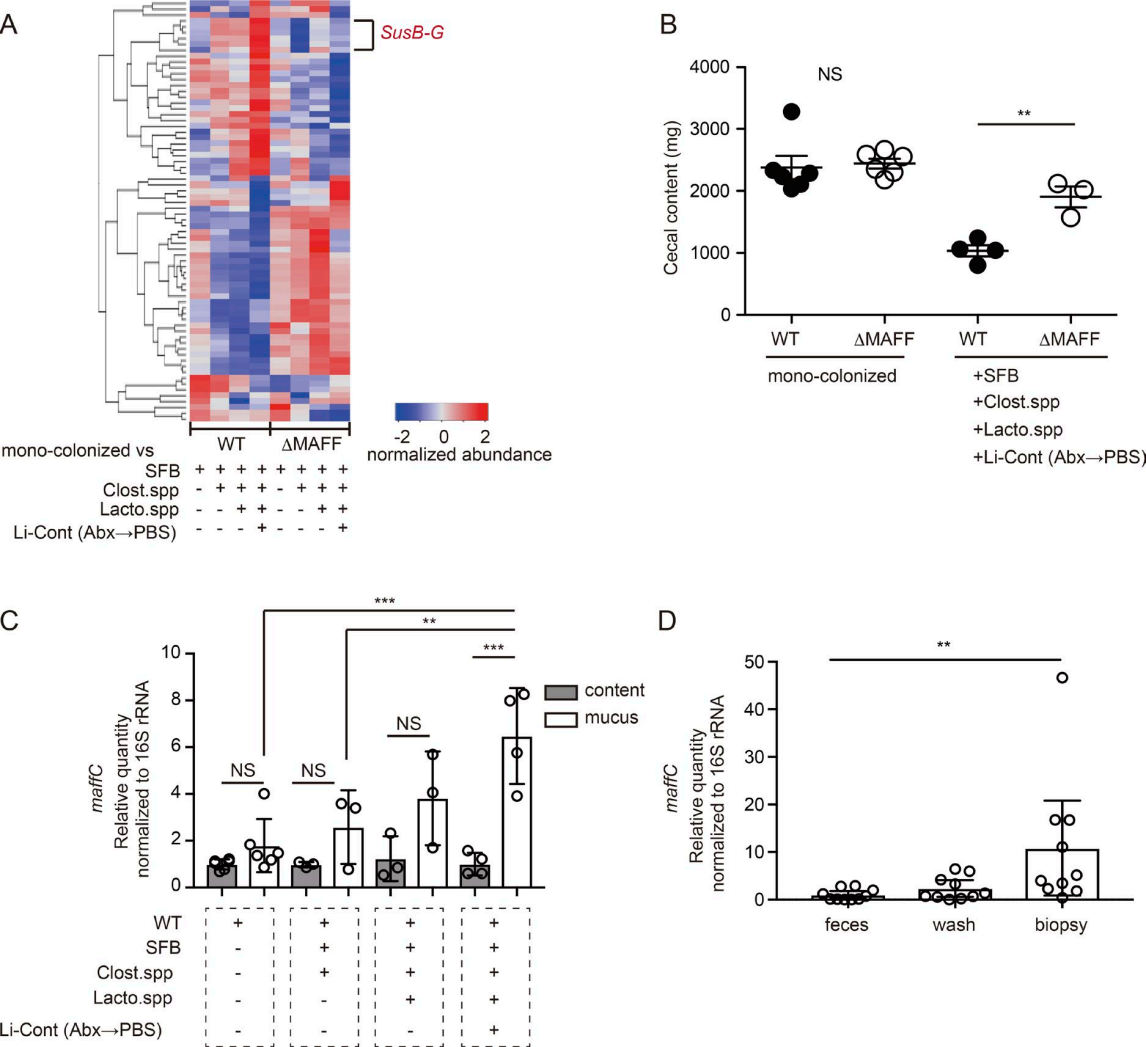
**MAFF system functions in colonic mucus of mice and humans with complex microbiota. (A)** Heat map analyses of BTWT and BTΔMAFF gene expression in indicated gnotobiotic conditions compared with monocolonization were performed with a likelihood ratio test. Each line shows the mean value of each group (*n* = 2–4 mice per group). The genes with a P-value adjusted <0.05 are shown. A full annotation of the heat map is shown in Table S3. **(B)** Cecal weight measured at the point of dissection of gnotobiotic mice. Each point represents an individual animal (*n* = 3–6 mice for each group). **(C)** qPCR analysis of *B. theta maffC* expression in large intestinal content and mucus collected from mono-colonized or gnotobiotic mice. Each point is data from an individual animal (*n* = 3–6 mice). **(D)** *B. theta maffC* expression in human feces, mucosal wash, and tissue biopsy samples from the large intestine. Each dot represents matched samples from one donor (*n* = 10 donors). Statistical analysis was performed with unpaired Student’s *t* tests (B), one-way ANOVA with Tukey’s multiple comparison test (C), and Kruskal-Wallis test with Dunn’s multiple comparison tests (D). **P < 0.01, ***P < 0.001. Error bars represent SEM.

The MAFF-dependent differential expression of conventional *SusB-G* genes after the addition of a diverse bacterial community indicated that *B. theta* acquired the capacity to use diet-derived starch via the MAFF system function (Fig. 6 A and Table S3). Indeed, the up-regulation of MAFF-dependent genes in *B. theta* inversely correlated with the weight of cecal contents, indicating more efficient consumption of diet-derived polysaccharides (Fig. 6 B). This symbiotic event occurred in the colonic mucus, as *maffC* expression by BTWT was observed specifically in this location but not in bacteria isolated from intestinal contents (Fig. 6 C). We analyzed samples from human gut to verify the regulation of *maffC* expression in a natural host of *B. theta* under homeostatic conditions. Consistent with the results from experimentally colonized mice, we observed that *B. theta maffC* expression was significantly higher in tissue biopsies compared with the fecal samples obtained from healthy donors (Fig. 6 D). These results suggest that the MAFF system cooperates with symbiotic partners to modulate the functional properties of *B. theta* within the mucus environment.

### IgA–MAFF axis induces Clostridiales expansion and regulates colonic homeostasis

**Figure 7.**
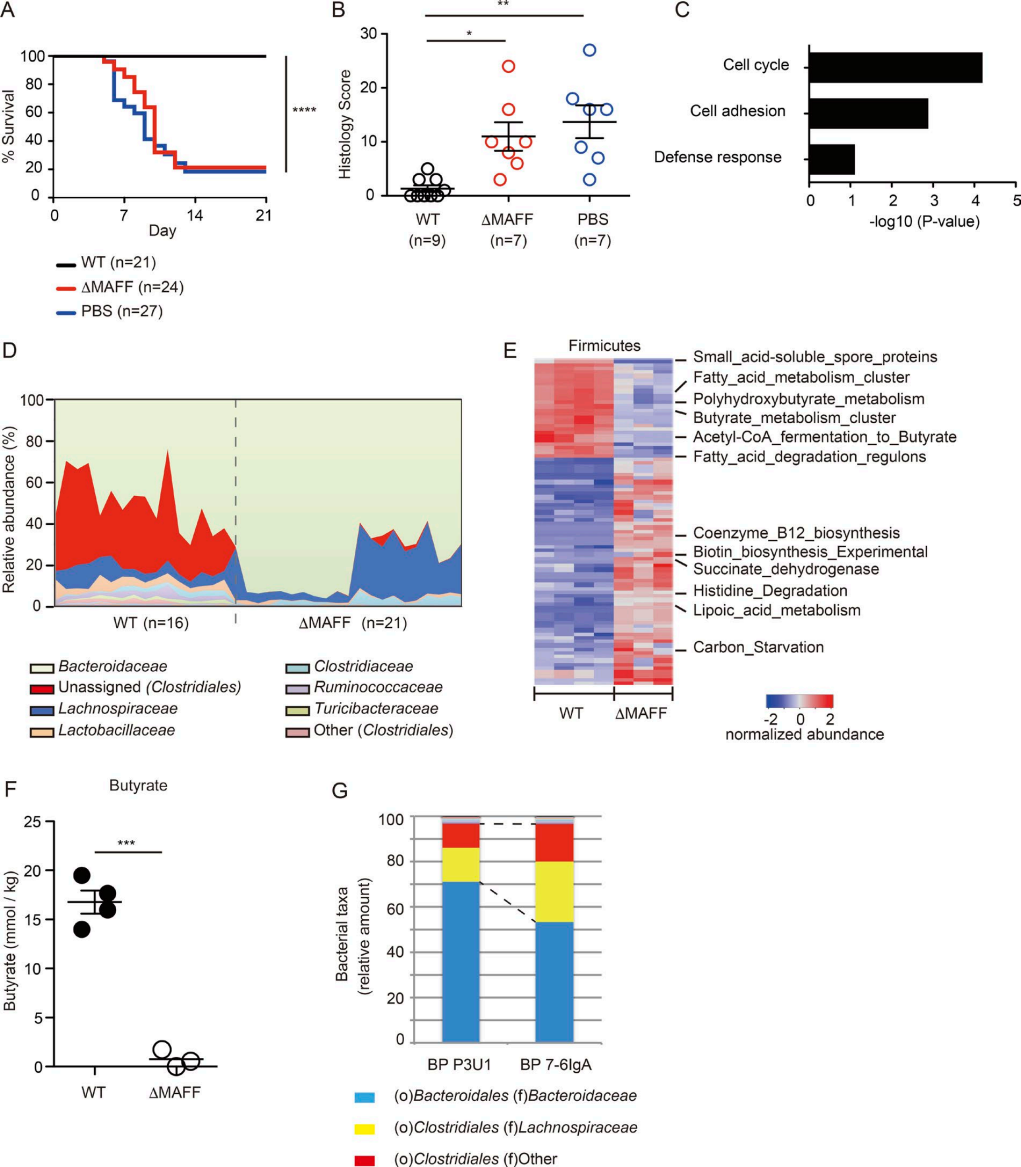
**MAFF induces expansion of Clostridiales and regulates colonic homeostasis.** Antibiotics-treated mice were inoculated with PBS, BTWT, or BTΔMAFF strains to generate SPF-Abx mice 4 wk before treatment with DSS. **(A)** Pooled Kaplan-Meier survival data of the five DSS colitis-sensitive experimental groups and matched PBS controls (*n* = 21–27 mice). All mice were treated with 4% DSS. **(B)** Blinded scoring of distal colon histology sections 10 d after initiation of DSS treatment (*n* = 7–9 mice). **(C)** DNA array was used to measure the gene expression profiles of PI^−^CD45^−^EpCAM^+^ colonic epithelial cells purified by FACS from the indicated SPF-Abx mice 4 wk after colonization. The differentially expressed genes (fold change > 2, P < 0.05, unpaired Student’s *t* test) were used to generate enriched pathways analyses, which were up-regulated in BTWT colonization (*n* = 4 mice) compared with BTΔMAFF strain (*n* = 4 mice). Full annotation is in Table S5. **(D and E)** The relative abundance of bacterial families identified with 16S rRNA analysis in feces (D; *n* = 16–21 mice per group) and RNA-Seq analysis of the Firmicutes gene expression in cecal samples of SPF-Abx mice collected at 4 wk after *B. theta* colonization (E), immediately before DSS treatment. The functional modules that aligned to the genome references of phylum Firmicutes and differentially expressed between BTWT (*n* = 4)- and BTΔMAFF (*n* = 3)-colonized mice are shown (log2 FoldChange>2, padj<0.05). Full annotation is in Table S4. **(F)** Concentrations of butyrate in cecal samples of SPF-Abx mice colonized with BTWT or BTΔMAFF strains measured by gas liquid chromatography (*n* = 3–4 mice for each group). **(G)** Gut microbial composition in cecal samples of BP mice, as in Fig. 3 A, was analyzed with 16S rRNA analysis and annotated with the taxonomic distribution at the family level. *n* = 5 mice for each group. Statistical analyses were performed with one-way ANOVA with Tukey’s multiple comparison test (B) or Student’s *t* test (F). *P < 0.05, **P < 0.01, ***P < 0.001, and ****P < 0.0001. Error bars represent SEM.

To explore whether the benefit conferred by MAFF expression by *B. theta* extends to the host, we used a chemical colitis model. We noted that dextran sodium sulfate (DSS)–sensitive and –resistant groups emerged in different batches of SPF-Abx mice with identical treatment (Fig. S4 A). Focusing on DSS-sensitive hosts, we found that colonization with BTWT, but not the BTΔMAFF strain, prevented the development of fulminant colitis (Fig. 7, A and B). Protection by BTWT correlated with enhanced expression of transcripts related to epithelial proliferation, cell adhesion, and defense mechanisms in the presence of BTWT compared with the BTΔMAFF strain (Fig. 7 C and Table S5). Robust proliferation of epithelial cells measured by Ki67 histology staining was confirmed following colonization with BTWT but not the BTΔMAFF strain (Fig. S4 B). These results suggest that the MAFF system enhances colonic epithelial regeneration.

We found that *B. theta* MAFF significantly altered the composition of the gut microbial community before DSS treatment. The colonization with BTWT but not the BTΔMAFF strain was associated with expansion of unassigned Clostridiales (30 ± 2.5% in BTWT versus 3.4 ± 1.18% in BTΔMAFF colonization group; P < 0.0001, *t* test; Fig. 7 D). In fact, the presence of Clostridiales strongly correlated with protection from colitis in the DSS-resistant groups mentioned above (49 ± 8.34% in DSS-resistant versus 3.4 ± 1.65% in DSS-sensitive groups; P < 0.0001, *t* test; Fig. S4 C). Surprisingly, MAFF expressed by *B. theta* also had a significant impact on gene expression of Firmicutes in SPF-Abx mice. Among the transcripts up-regulated by the functional MAFF system in *B. theta* were those related to fatty acid metabolism, especially those for butyrate production (Fig. 7 E and Table S4). Indeed, BTWT but not the BTΔMAFF strain increased the concentration of cecal butyrate, a well-known epithelial protective factor that has been shown to be largely produced by Firmicutes (Macfarlane and Macfarlane, 2003; Maslowski et al., 2009; Plöger et al., 2012; Fig. 7 F). Deletion of MAFF in *B. vulgatus* was also associated with the altered composition of Firmicutes, suggesting a conserved interphylum functional role of the MAFF system in Bacteroidetes, modulating the expansion and function of species belonging to Firmicutes (Fig. S4 D). Finally, IgA appeared to play a critical role in this symbiotic interphylum partnership; as in the presence of hybridoma secreting 7-6IgA in the backpack *Rag1^−/−^* experiments, *B. theta* drove the expansion of Firmicutes, especially Clostridiales (29.2 ± 0.02% in BP P3U1 versus 44.9 ± 0.02% in BP 7-6IgA; P < 0.001, *t* test; Fig. 7 G).

## Discussion

**Figure 8.**
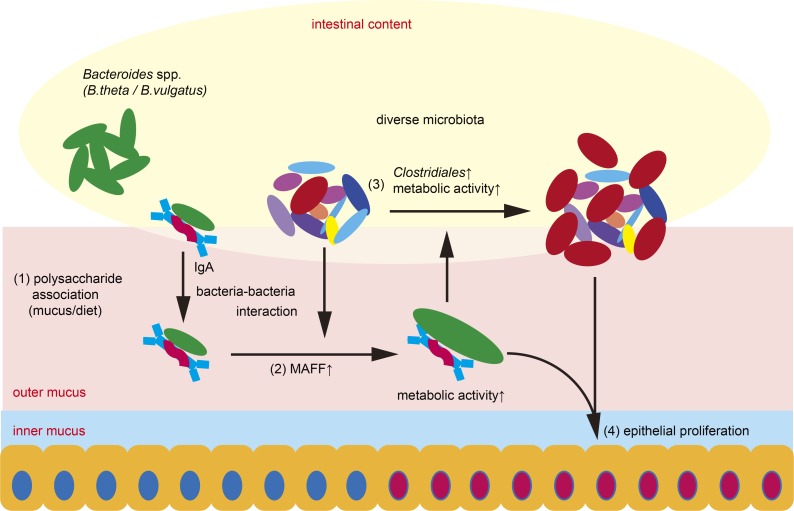
**Summary scheme for IgA–MAFF axis in the colon.** Optimal in vivo metabolic capacity of *B. theta* requires IgA and MAFF. (1) IgA binds to *B. theta* via glycan–glycan interactions (IgA-glycan and LPS) and enhances the association of the bacteria with host mucus and/or diet-derived polysaccharide. (2) Mucus-associated *B. theta* induces MAFF system expression dependent on interaction with Firmicutes members such as Clostridiales and enhances the metabolic activity of *B. theta*. (3) Metabolically active *B. theta* induces the expansion of Clostridiales members, altering the composition of the gut microbial community. (4) The MAFF-induced microbiota rich in Clostridiales enhances proliferation and regeneration of colonic epithelial cells during DSS colitis. MAFF in *B. vulgatus* shows similar effects. Mucus-specific MAFF expression was also observed in human colon biopsies, suggesting that the MAFF system is involved in the maintenance of colonic homeostasis in both mice and humans.

Mucosal IgA is not only produced in response to infections in order to eliminate the pathogens, but is also constantly generated in response to commensal bacterial communities. Although several pathways of IgA generation have been defined, and we are beginning to appreciate some functions for IgA beyond pathogen exclusion, the mechanisms by which this immunoglobulin regulates microbial communities is still largely unknown. For example, whether polyreactive and/or bacteria-nonspecific IgA (bystander IgA) present in the intestine shape the composition of the gut microbiota is insufficiently explored. In addition, it was not clear if the many post-translational modifications that characterize secretory IgA contribute to its homeostatic functions. This study suggests a novel pathway by which IgA might shape the microbiome and regulate colonic homeostasis (Fig. 8). The initial step involves glycan-dependent binding of IgA to surface bacterial components, such as LPS of gram-negative bacteria like *B. theta*. The in vivo expression patterns of *B. theta maffC/D* indicate that (a) the binding of bystander IgA can modulate gene expression of *B. theta* in the colonic mucus; (b) diverse bacterial species, especially those belonging to Clostridiales, are essential for the expression and function of the MAFF system; and (c) MAFF is highly expressed in the colonic mucus of humans, a natural host of *B. theta*. These data suggest that IgA, mucus, and a diverse microbial community provide signals that influence how *B. theta* adapts to its homeostatic niche in the steady state.

The MAFF-dependent interaction between the two major Bacteroidetes–Firmicutes phyla induces a dynamic change of the gene expression profiles of both bacteria, up-regulating the polysaccharide utilization activity of *B. theta* and stimulating the fatty acid utilization and expansion of butyrate-producing Clostridiales. It remains to be determined whether MAFF expression by *B. theta* also affects the replication rates of adjacent bacteria that synthesize these beneficial metabolites. Globally, these metabolic changes may directly stimulate the proliferation and regeneration of colonic epithelial cells in response to epithelial damage. Thus, the IgA–MAFF axis plays a critical role regulating the composition and metabolic activity of the complex microbial community, thereby promoting colonic homeostasis.

During the revision of this manuscript, Donaldson et al. (2018) reported that IgA coating enhanced intestinal colonization of *B. fragilis* by increasing adherence of this bacterium to epithelial cells. The capsular polysaccharides of *B. fragilis* mediate binding to IgA, enhancing colonization stability in monocolonized mice. Our study confirms and extends this view by showing that IgA is not only altering the gene expression of mucus-associated, IgA-coated bacteria, but is also an essential component of the regulatory network modulating interphylum bacteria interaction. Within this network, the MAFF system plays an essential role downstream of utilization of mucus and/or diet-derived polysaccharides by Bacteroidetes aided by the presence of Firmicutes and IgA, thereby regulating the composition and metabolic function of the whole gut microbiome.

There are many unanswered questions, for which future studies will hopefully provide the answers. For example, what exactly are the mechanisms through which the MAFF system performs its role, including how *maffC* and *maffD* cooperate to confer a competitive advantage in vivo? The identification of molecular targets of putative *maffC* transporter activity also remains to be elucidated. It is possible *B. theta* directly senses Clostridiales-derived metabolites, or alternatively responds to the depletion of local nutrients that indicates other bacteria are competing for finite local supplies within the mucosal niche. In addition, the mechanism for regulation of MAFF-related genes such as BT2264/BT2263 (Glenwright et al., 2017) is an important issue for the future study. The genetic structure surrounding MAFF among Bacteroidales contains another *SusC/D* pair (i.e., BT2264/BT2263) in a reverse orientation (Fig. 3 D). BT2267 was previously reported to be a homologue of a DNA invertase found in *B. fragilis*, which regulates the expressions of multiple genes in an on–off manner (Nakayama-Imaohji et al., 2009). Interestingly, we observed that the expressions of BT2264/BT2263 were significantly decreased in backpack animals secreting 7-6IgA, while those of BT2268/BT2269 (*maffC/D*) were significantly increased. One possible explanation for this phenomenon is that BT2267 induced DNA inversion of the promoter sequence of BT2264/BT2263 and suppressed their expression. It would be interesting to investigate the in vivo function of the BT2264/BT2263 pair and how it complements the MAFF system.

Our study highlights the critical effect of microbial interactions for the maintenance of colonic homeostasis and the importance of host immune elements in contributing to this balance. The impact of microbial diversity on metabolic fitness of individual species in vivo should be a consideration in future experiments addressing the composition and function of endosymbiont populations.

## Materials and methods

### Mice and colonization experiments

C57BL/6 WT mice were purchased from CLEA Japan Inc. OTII-Tg, CD3ε^−/−^, and *Rag1*^−/−^ on the C57BL/6 background were bred in house. All animals were maintained in SPF conditions at the Institute of Laboratory Animals, Graduate School of Medicine, Kyoto University. 8-wk-old males were used for experiments. For antibiotic treatment, the mice were given 100 mg/kg metronidazole by oral gavage every 24 h for 7 d, in addition to 0.66 mg/ml ciprofloxacin (Wako) and 10 mg/ml of artificial sweetener (Pal Sweet; Ajinomoto) administered in the drinking water (Bloom et al., 2011; Lee et al., 2013). For colonization experiments, mice were gavaged with 3% NaHCO_3_/PBS with or without 1 × 10^8^ CFU of cultured bacteria 2 d after antibiotic withdrawal. All antibiotics-treated mice were maintained in an individual ventilation system and transferred to fresh cages twice per week. To measure colonization efficacy, fecal samples were homogenized, serially diluted in PBS, and plated on selective media containing erythromycin (10 μg/ml) or chloramphenicol (15 μg/ml). The mucus-adherent bacteria fraction was harvested by gently scraping the mucosal surface with a coverslip after rinsing twice with PBS to remove residual luminal content. Matched, collected luminal contents were used as the control for CFU analysis. For monocolonization and gnotobiotic experiments, GF C57BL/6 mice were generated and maintained at Sankyo Laboratories Japan or the Johns Hopkins University animal facility (monocolonization) or at the RIKEN Center for Integrative Medical Sciences (gnotobiotic mice with serial colonizations). SFB colonization of GF mice was performed as described previously (Umesaki et al., 1995). To create a stock of Bacteroidales-depleted SPF colonic bacteria for oral gnotobiotic mouse colonization, the contents of the large intestine were collected from control antibiotics–treated SPF mice given PBS gavage (Li-cont (Abx→PBS)), 4 wk after cessation of antibiotics. The intestinal contents of four Abx→PBS mice were pooled to create an inoculum for each gnotobiotic experimental group. Sample size was determined based on published studies. The animal cages that exhibited severe fighting were excluded from the analysis. All experiments were performed in accordance with the approved protocols from the Institute of Laboratory Animals at Kyoto University, the Institutional Animal Care at RIKEN, and the Animal Care and Use Committee at Johns Hopkins University.

### Human study

The protocol for donor recruitment, informed consent, collecting the anonymous samples, and acquiring data conformed to the Declaration of Helsinki and was approved by the Research Ethics Committee of RIKEN Yokohama Branch and the Institutional Review Boards at Kyoto University Hospital. Samples were collected from 10 donors (age 36–80, average 63.6 yr old, seven males and three females) at the Kyoto University Hospital. The donors were healthy or had small colonic polyps that could be treated with endoscopic polypectomy. Fecal samples were collected a day before or during the pretreatment of colonoscopies and kept at 4°C for a maximum of 2 h before storage at −80°C. During colonoscopies, a healthy region of the distal colon mucosal surface was washed with saline, and ∼300 ml of washed solutions was collected by aspiration. The washed solutions were immediately centrifuged, and the bacterial pellets were treated with RNAprotect Bacteria Reagent (QIAGEN) and stored at −80°C. Biopsy samples were obtained after the washing steps and immediately submerged in RNAlater solution. Total RNA samples were extracted from feces using ZR Soil/Fecal RNA MicroPrep (Zymo Research) or from the pellets of mucosal washes and biopsies using RNeasy (QIAGEN). cDNA synthesis and quantitative PCR (qPCR) were performed as described in the qRT-PCR section below.

### Hybridoma cells, monoclonal antibodies, and backpack animals

Monoclonal IgA was purified from hybridoma as described previously (Peterson et al., 2007). To induce mucosal OVA-specific antibodies, OTII-Tg CD4^+^ T cells were enriched by magnetic sorting (CD4^+^ T Cell Isolation Kit; Miltenyi Biotec) from the spleen and peripheral lymph nodes, and 1 × 10^6^ cells were intravenously transferred into CD3ε^−/−^ mice. Recipient CD3ε^−/−^ mice and C57BL6 controls were continuously given 1 mg/ml OVA (Sigma) in drinking water. After 6 wk, a single-cell suspension was prepared from small intestinal lamina propria and fused with P3U1 myeloma cells by adding 50% polyethylene glycol in RPMI. Hybridoma clones producing OVA-specific IgA (clone 7-6) and IgG (clone 76-3) were selected based on the screening of culture supernatants by ELISA using OVA-coated plates. 7-6IgA and 76-3IgG hybridoma cell lines were injected into nude mice, and monoclonal antibodies were purified from ascites with Protein L (7-6IgA) or Protein A (76-3IgG). To generate backpack animals, hybridoma cells or P3U1 myeloma cells were grown in DMEM and washed twice in PBS, and then 2 × 10^6^ cultured cells in 0.3 ml of PBS were injected subcutaneously beneath the dorsal skin of *Rag1*^−/−^ recipients.

### DSS-induced colitis and histology

Mice were treated with 3–4% wt/vol DSS (molecular weight, 36,000-50,000; MP Biochemicals) in drinking water for 7 d. At the time of dissection, the colonic tissues were opened longitudinally, fixed in 10% buffered formalin, and embedded in paraffin, and 5-μm sections were stained with hematoxylin and eosin. Histological scoring was performed by a pathologist in a blinded fashion, as previously described (Morteau et al., 2000). Three histological parameters were evaluated using semiquantitative scoring systems: (1) severity of inflammation (0–3: none to severe); (2) depth of injury (0–3: none to transmural); and (3) crypt damage (0–4: none to entire crypt damaged). The score of each parameter was multiplied by a factor reflecting the area of involvement (1–4: 0–25%, 26–50%, 51–75%, 76–100%) to give a cumulative score.

### Glycan analysis of the monoclonal antibodies

Periodic acid-Schiff staining of purified antibodies was done using SDS-PAGE with a glycoprotein staining kit (Thermo Fisher Scientific Pierce) following the manufacturer’s instructions. Equal protein loading was confirmed by SDS-PAGE followed by immunoblot analysis with HRP-conjugated goat anti-mouse IgA (Bethyl). Chemiluminescence signal was detected using a Luminescent Image Analyzer LAS-4000 (Fuji). The amounts of total carbohydrate on purified IgA clones were determined by using the Total Carbohydrate Colorimetric Assay Kit (BioVision). Lectin microarray was performed as described (Inoue et al., 2013). Briefly, protein was labeled with Cy3 and then applied to lectin array (LecChip; GP Biosciences). The lectin signals were measured with GlycoStation Reader 1200. Scanned images were analyzed with Array-Pro Analyzer (Media Cybernetics) and GlycoStation Tools (GP Biosciences). Lectin concentration was normalized to the total protein concentration measured with a Micro BCA Protein Assay Kit (Thermo Fisher Scientific Pierce).

### Bacterial strains, plasmids, and culture conditions

Bacterial strains and plasmids are summarized in Table S6. Bacterial strains other than *Escherichia coli* were cultured anaerobically at 35°C in liquid Gifu anaerobic medium (GAM; Nissui Pharmaceutical) or on GAM agar supplemented with 0.2% glucose. To isolate laboratory strains, cecal content from SPF mice was quickly plated on GAM agar plates and incubated anaerobically for 2 d. Isolated colonies were identified by 16S ribosomal RNA (rRNA) sequencing. *E. coli* S17-1 λ pir strain was aerobically grown in Luria-Bertani medium at 37°C. Antibiotics were added at the following concentrations when required: erythromycin (10 μg/ml), chloramphenicol (15 μg/ml), and gentamicin (200 μg/ml). In minimal medium culture, 0.5% glucose or purified 7-6IgA was used as the carbon source. Bacterial growth was measured with a BioPhotometer plus (Eppendorf) at OD 600.

### Flow cytometry

To assess bacteria–antibody interactions, 3 × 10^7^ cultured bacteria were incubated with various concentrations of 7-6IgA or 76-3IgG in a final volume of 200 μl in PBS for 1 h at 37°C. After washing with PBS, bacteria were stained with FITC or Alexa647 (Molecular Probes)-conjugated anti-mouse IgA (Southern Biotech), anti-mouse IgG1 (Clone A85-1; BD), and SybrGreen (Molecular Probes). For OVA-IgA inhibition assay, various amounts of 7-6IgA were preincubated with 10× volume of OVA (Sigma) at 37°C for 0.5 h, and then the 7-6IgA–OVA immune complexes were incubated with *B. theta* at 37°C for 1 h. For the 7-6IgA LPS binding assay, cultured, untreated *B. theta* (live) was heat killed (70°C, 30 min) or fixed with 70% ethanol (room temperature, 15 min), washed with PBS, and incubated with 7-6IgA and secondary antibodies as above. All samples were acquired on LSRFortessa (BD Biosciences), and the data were analyzed with FlowJo software (v9.3.2 and v10.0.6; TreeStar).

### Generation of deletion mutants

Mutant strains of *B. theta* and *B. vulgatus* were generated as previously described (Lee et al., 2013). Briefly, left DNA segments and right DNA segments flanking the target region were PCR amplified and fused. The fused PCR product was cloned into the BamHI and XbaI site of the *Bacteroides* conjugal suicide vector pKNOCK*-bla-ermGb* and conjugated into *B. theta*. Colonies were selected for erythromycin resistance and passaged for 5 d and then spread onto GAM agar plates. The resulting colonies were screened with replica plating to detect the desired mutants. The same strategy was used to generate deletion mutants of *B. vulgatus*. Complementation of the MAFF deletion mutant was accomplished by PCR amplification of the BT1311 promoter and MAFF genes (BT2268/2269), including the putative BT2268 promoter. These fragments were fused and cloned as a BamHI and XbaI fragment into p*NBU2-bla-ermGb*. The list of primers used is shown in Table S7.

### FISH

Colonic tissues were fixed in Carnoy’s solution (Wako) to preserve the mucus layer, embedded in paraffin, and cut to 5 µm. The sections were hybridized to a mixture of three Bacteroidetes-specific probes labeled with Cy3. The sequences of the probes are (1) 5′-CATTTGCCTTGCGGCTA-3′ (Momose et al., 2011), (2) 5′-CCAATGTGGGGGACCTT-3′ (Manz et al., 1996), and (3) 5′-AGCTGCCTTCGCAATCGG-3′ (Weller et al., 2000). Since phylum Bacteroidetes was efficiently depleted by metronidazole and ciprofloxacin (Bloom et al., 2011; Fig. S4 C), this strategy specifically visualized *B. theta* in SPF-Abx mice. Images were acquired on a Zeiss LSM 710 confocal laser-scanning microscope. The bacterial length was measured with Adobe Photoshop CS 5.1 on acquired images.

### Total RNA extraction from bacteria

Total RNA was extracted from bacterial cultures using RNAprotect Bacteria Reagent and RNeasy kit (QIAGEN) or from intestinal contents using ZR Soil/Fecal RNA MicroPrep (Zymo Research). Total RNA of mucus-attaching bacteria was isolated using RNAprotect Bacteria Reagent and RNeasy kit.

### qRT-PCR

Total RNA was subjected to DNase treatment (Invitrogen), and RT was performed using a SuperScript VILO Master Mix (Invitrogen). qRT-PCR analysis was performed using Thunderbird SYBR qPCR mix (Toyobo) and the Lightcycler 480 apparatus (Roche). Gene-specific primers are described in Table S7 (Odamaki et al., 2008; Tong et al., 2011).

### RNA-Seq sample preparation

Total RNA was subjected to DNase digestion (Baseline-ZERO; Epicentre) and passed through a MEGAclear column (Ambion) to deplete RNAs <200 nt (removing most 5S rRNA and transfer RNA). The samples were subjected to another round of DNase digestion (DNA-free TURBO; Ambion), passed through another MEGAclear column, and finally subjected to bacterial rRNA depletion (Ribo-Zero Magnetic Kit Bacteria; Epicentre). PCR with universal 16S rRNA gene primers was used to verify the absence of detectable genomic DNA in each RNA preparation. The concentration and purification of bacterial mRNA were measured using Bioanalyzer 2100 (Agilent). Double-stranded cDNA was synthesized by SuperScript II (Invitrogen), and then the library was prepared using a TruSeq Stranded RNA HT Kit (Illumina) and quantified by qRT-PCR according to the Illumina protocol. Libraries were sequenced using the Illumina HiSeq platform.

### Analysis of RNA-Seq data

To generate *B. theta* gene expression profiles, the raw Illumina reads were trimmed with fqtrim (http://ccb.jhu.edu/software/fqtrim/index.shtml) with the parameters “-A –D –q 20 –l 50.” The trimmed reads were then mapped to a combined microbiome-host genome database using BWA-MEM (version 0.7.12-r1039; Li, 2013 *Preprint*). To generate the reference database, the combined microbiome reference genome database (1,253 species of bacteria, 97 species of archaea, 326 species of lower eukaryotes, and 1,420 species of viruses) was downloaded from the Human Microbiome Project site (Martin et al., 2012) and concatenated with the complete sequence sets of mouse genome database (GRCm38/mm10: Dec. 2011; downloaded at the UCSC Genome Bioinformatics site, http://genome.ucsc.edu/). The annotation data of *B. theta* were downloaded from Ensembl gene annotations (Kersey et al., 2016). The sequences that mapped to coding regions of the *B. theta* genome were counted using HTSeq Count (Anders et al., 2015). For the functional abundance profiles of Firmicutes, the raw reads were uploaded to MG-RAST (Meyer et al., 2008) and processed by default settings, and the count of the functional annotations was retrieved at level 3 of SEED Subsystems with the script of “mg-compare-functions.py,” described in the MG-RAST application programming interface (Wilke et al., 2015). The counting data of HTSeq Count or MG-RAST were analyzed using DESeq2 version 1.8.1 (Love et al., 2014), run in R version 3.2.2.

### 16S rRNA gene sequencing and analysis

Genomic DNA was isolated from fecal samples using the PowerSoil bacterial DNA extraction kit (MoBio). DNA samples were amplified using the following barcoded primers targeting the V1–V2 region of the 16S rRNA gene (primers used are listed in Table S7; Turner et al., 1999). The amplicons were purified with AMPure XP beads (Beckman); indices and sequencing adaptors were introduced using the Nextera XT Index kit (Illumina), quantified by using Thunderbird SYBR qPCR mix (Toyobo), and pooled according to the Illumina protocol. Prepared library was sequenced with the Illumina MiSeq platform. Reads were then processed as previously described (Pylro et al., 2014). Briefly, the USEARCH algorithm was used to filter the high quality reads, cluster into operational taxonomic units (OTUs), and remove chimeras based on the database in RDP Classifier (Edgar, 2010; Edgar et al., 2011). Minimum identity of 97% was set to identify OTUs. The QIIME scripts (version 1.9.1) were then used to assign taxonomy, align sequences to the Greengenes reference database, perform α and β diversity analysis, and summarize taxonomy (Caporaso et al., 2010).

### DNA array analysis

To isolate intestinal epithelial cells, colonic tissues were incubated with 1 mM EDTA in PBS at 37°C for 20 min, and the single-cell suspensions were prepared by mechanical dissociation with vigorous shaking in PBS. The suspensions of epithelial cells were stained with anti-CD45 (30-F11) and anti-EpCAM (G8.8; both from BioLegend), followed by propidium iodide (PI). PI^−^CD45^−^EpCAM^+^ fraction was sorted to 90–95% purity with BD Aria III by using a 130-μm nozzle, and RNA was isolated using the RNeasy kit (QIAGEN). cDNA was amplified using a NuGen WT Ovation Pico kit and hybridized to an Affymetrix GeneChip (Mouse Genome 430 2.0 Array). The data were analyzed with GeneSpring12.5 (Agilent) and DAVID (Database for Annotation, Visualization, and Integrated Discovery; Huang et al., 2009).

### Data availability

RNA-Seq, 16S rRNA, and microarray data are deposited in the Gene Expression Omnibus (GEO) under accession no. GSE115814, GSE115902, and GSE97504.

### Immunohistochemistry

Colonic tissues were fixed in 10% formalin (Wako) before paraffin embedding. 5-μm sections were incubated with anti-Ki67 (Abcam). Slides were washed and incubated with HRP-labeled anti-rabbit (EnVision+System; Dako), followed by diaminobenzidine (Vector) and weak counterstaining with diluted hematoxylin (Wako). Images were acquired on a Zeiss AxioImager.A2 microscope.

### ELISA

Fecal samples were collected from mice and resuspended in sterile PBS at a concentration of 100 mg/ml by weight. Supernatants were collected and stored at −30°C until use. IgA concentration was determined using mouse IgA ELISA Quantitation Set (Bethyl). For the binding inhibition assay, purified 7-6IgA was incubated with various amounts of OVA for 30 min at room temperature and then incubated with OVA-coated plates (coat 1 h with 100 μg/ml OVA/PBS at room temperature). OVA-bound IgA was detected with mouse IgA ELISA Quantitation Set. For the LPS-IgA binding assay, LPS was purified from 2 × 10^9^ *B. theta* cultured in MM-G using the LPS Extraction kit (iNtRON Biotechnology, Inc.) and then biotinylated using EZ-link Hydrazide-LC-Biotins (Thermo Scientific). MaxiSorp Nunc-Immunoplates (Thermo Scientific) were coated by anti-mouse IgA (Bethyl), and the equal amounts of monoclonal mouse IgA proteins (clones M18-254 from BD, clone S107 from eBioscience, and clone 7-6) were added. The captured IgA clones were incubated with biotinylated *B. theta* LPS, and the amounts of IgA-bound LPS were detected with Streptavidin-HRP and chromogenic substrate tetramethylbenzidine (Life Technologies).

### Measurement of butyrate

Butyrate concentrations were determined in cecal contents using gas liquid chromatography as described previously (Tsukahara et al., 2014).

### Statistics

We tested normal distribution of the data and applied parametric or nonparametric tests according to the experiment. Two-tailed Student’s *t* tests were employed for comparison of two groups. For multiple comparisons with more than two groups, one-way ANOVA with Tukey’s multiple comparison tests or Wilcoxon test with Dunn’s multiple comparison tests were used. For comparisons of RNA-Seq data of gut microbiota, likelihood ratio tests were employed, and adjusted P-values were used to determine significance.

### Online supplemental material

Fig. S1 describes the characteristics of fecal IgA of OTII→CD3ε^−/−^ mice and the protein profile of purified monoclonal antibodies. Fig. S2 shows a phylogenetic tree of BT2268 (*maffC*) homologues. Fig. S3 shows the additional in vitro and in vivo features of MAFF deletion mutants of *B. theta* and *B. vulgatus*. Fig. S4 shows the in vivo effect of antibiotic treatment and MAFF molecule for the maintenance of colonic homeostasis. Table S1 provides the summary of RNA-Seq reads. Table S2 provides a full set of differentially expressed *B. theta* genes in cecal contents of backpack animals (BP 7-6 versus BP P3U1). Table S3 gives the full annotation of differentially expressed *B. theta* genes of BTWT and BTΔMAFF in gnotobiotic mice (Fig. 6 A). Table S4 gives the full annotation of differentially expressed functions of phylum Firmicutes in cecal contents of antibiotics-treated SPF animals colonized with BTWT or BTΔMAFF (Fig. 7 E). Table S5 gives a full set of up-regulated genes in colonic epithelial cells of antibiotics-treated SPF animals colonized with BTWT compared with BTΔMAFF-colonized mice (Fig. 7 C). Table S6 provides the bacterial strains and plasmids used in this study. Table S7 provides sequences of primers used in this study.

## Supplementary Material

Supplemental Materials (PDF)

Tables S1-S7 (Excel file)
