# Supplementary material for: IgA regulates the composition and metabolic function of gut microbiota by promoting symbiosis between bacteria
Source: J Exp Med. 2018 Aug 6;215(8):2019–34. doi: 10.1084/jem.20180427 (PMC6080902; doi:10.1084/jem.20180427)
Supplement: Supplemental Materials (PDF) [file JEM_20180427_sm.pdf]

Supplemental material

Nakajima et al., https://doi.org/10.1084/jem.20180427

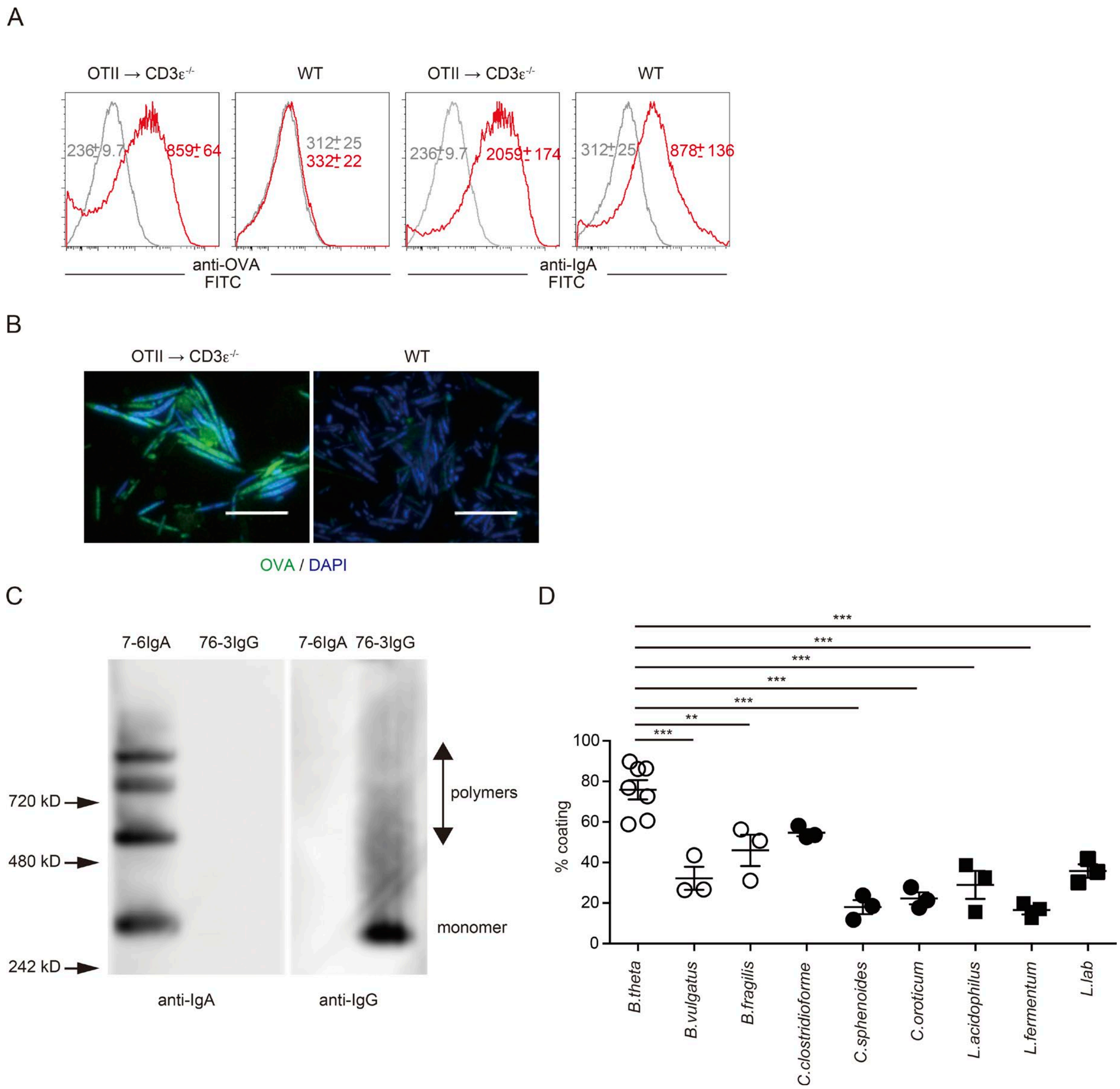

Figure S1. **Anti-OVA IgA nonspecifically bound on bacterial surfaces. (A)** Fecal samples were collected from OTII→CD3ε$^{-/-}$ mice at 4 wk of OVA treatment, and bacterial cells were enriched with a single Percoll gradient procedure (20% versus 80%). The bacterial cells were stained for surface-bound OVA and IgA (red histograms) or remained unstained (gray histograms). The numbers on histograms indicate the summary of geometric mean fluorescence intensity with standard error from four independent experiments. **(B)** 100 mg OVA was orally given bolus to OTII→CD3ε$^{-/-}$ mice at 4 wk of OVA treatment or to nontreated WT mice. The cecal contents were collected after 16 h of gavage and stained for anti-OVA–Alexa488 and DAPI. Representative images of three independent experiments are shown. Scale bars, 10 µm. **(C)** Immunoblots of purified 7-6IgA or 76-3IgG. The samples were run on a nonreducing polyacrylamide gel, transferred to nitrocellulose, and immunostained with anti-IgA or anti-IgG. **(D)** Cultured bacterial strains were incubated with the fecal extract of OTII→CD3ε$^{-/-}$ mice containing 250 µg/ml of total IgA, and the proportion of IgA-coated bacteria was measured by flow cytometry. n = 3–7 independent experiments for each group. Statistical analyses were performed with one-way ANOVA with Tukey's multiple comparison test. **P < 0.01 and ***P < 0.001. Error bars represent SEM.

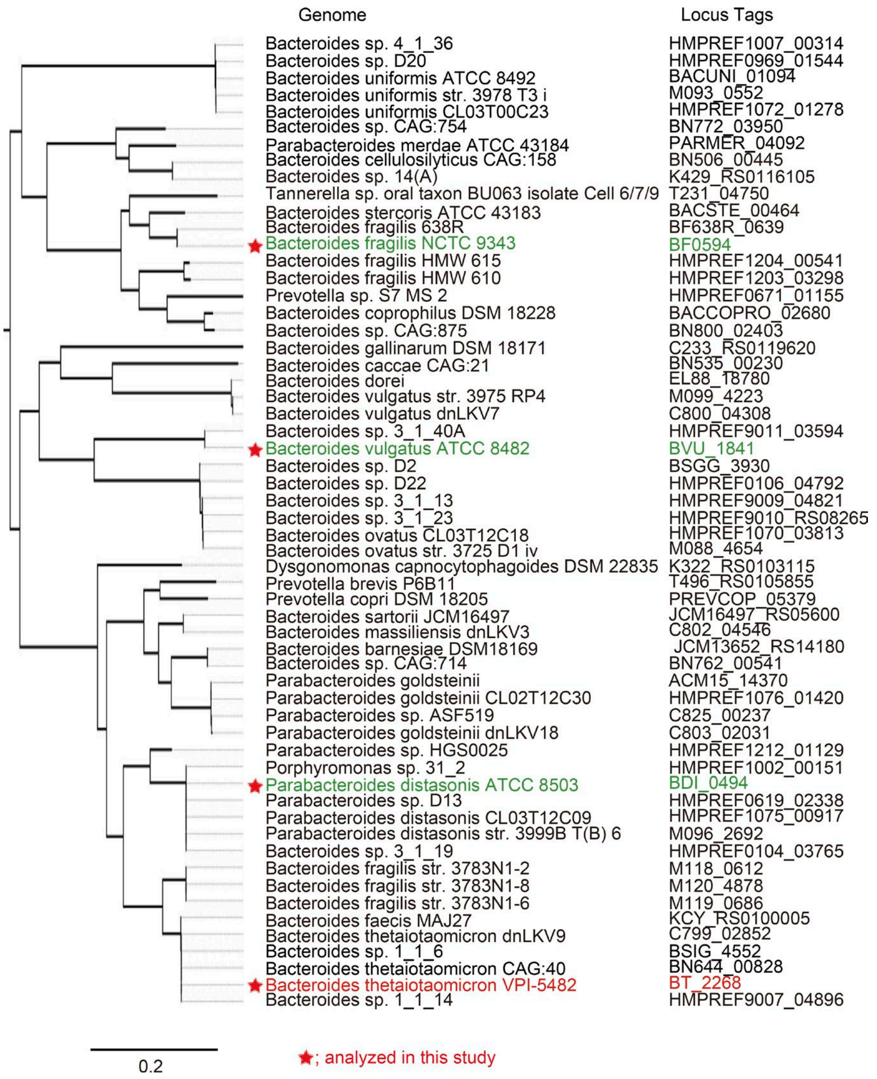

Figure S2. **A phylogenetic tree of BT2268 homologues.** Amino acid sequences of BT2268 were used to query sequenced genomes using BLAST. Hits with scoring matrix = PAM30 and score = >600 bits were examined for resemblance to BT2268. The obtained amino acid sequences were concatenated and locally aligned using MUSCLE (Edgar, 2004). The tree was constructed using RAxML (Stamatakis, 2014) and the neighbor-joining method with a bootstrap of 100 replicates. Asterisks indicate the bacterial strains chosen for further analyses in this study.

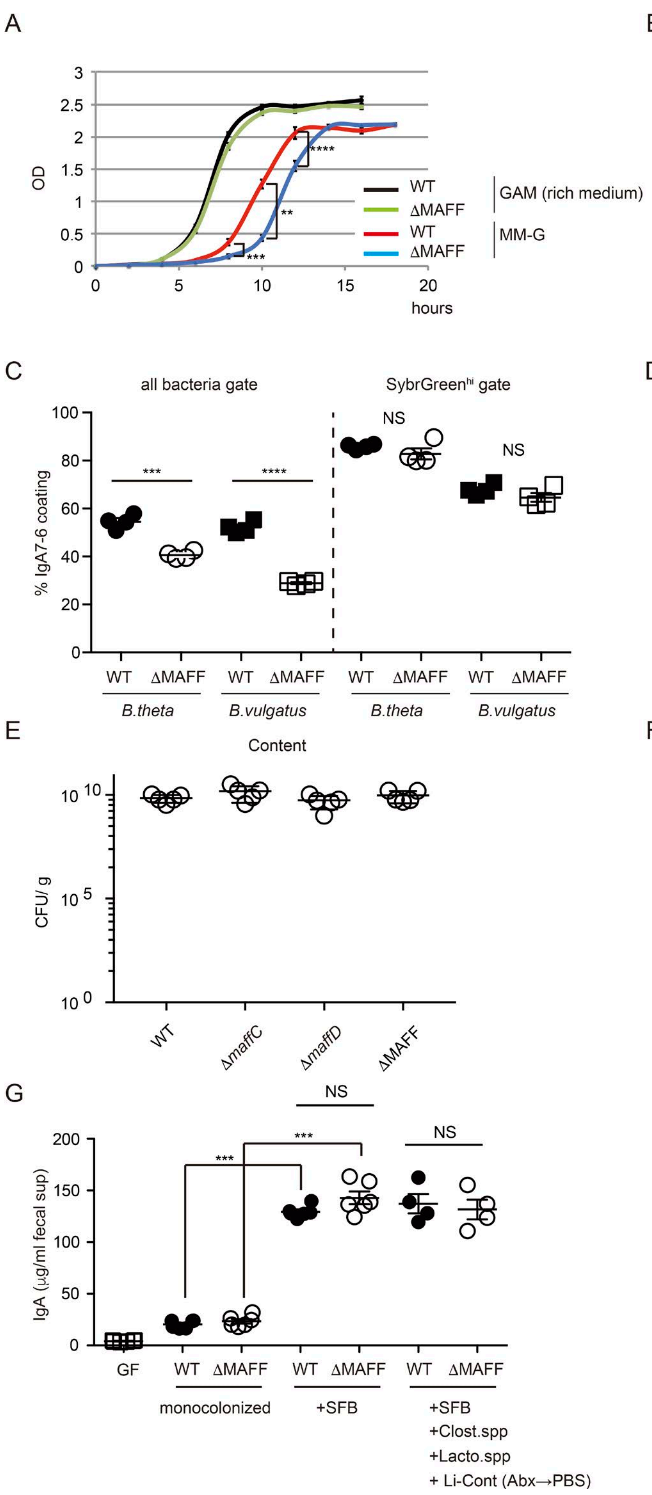

Figure S3. **In vitro and in vivo features of WT and ΔMAFF strains. (A)** The growth curves of BTWT and BTΔMAFF strains in nutrient-rich Gifu anaerobic medium (GAM) and in MM-G. *n* = 3 independent cultures. **(B)** The growth of indicated bacterial strains cultured in MM-G was compared at the log phase of growth (*n* = 3 independent cultures for each group). **(C)** Indicated bacterial strains were double stained with SybrGreen and 7-6IgA and analyzed by flow cytometry. The percentages of 7-6IgA–coated cells in the total bacteria population (all bacteria gate) and in the SybrGreen^{hi}-gated populations (SybrGreen^{hi} gate) are shown (*n* = 4 independent cultures for each group). **(D)** BTWT and BTΔMAFF strains were cultured in minimal medium supplemented with 7-6IgA as the sole carbon source at a concentration of 9,500 µg/ml and measured for OD = 600 absorption at the indicated time points (*n* = 1 for each group). **(E and F)** Antibiotics-treated SPF mice (SPF-Abx mice) were gavaged with 1 × 10^8 of the indicated WT or mutant strains of chloramphenicol-resistant (Crm^r) *B. theta*. The intestinal content (E) and mucus (F) were collected on day 15 of colonization, and the CFUs of *B. theta* were measured by selective plating (*n* = 4–5 mice for each group). **(G)** ELISA measurement of the fecal IgA concentration in gnotobiotic mice as depicted in Fig. 5 C (*n* = 4–6 mice for each group). Statistical analyses were performed with Student's *t* test (A) or one-way ANOVA with Tukey's multiple comparison test (B, C, and G). **P < 0.01, ***P < 0.001, and ****P < 0.0001. Error bars represent SEM.

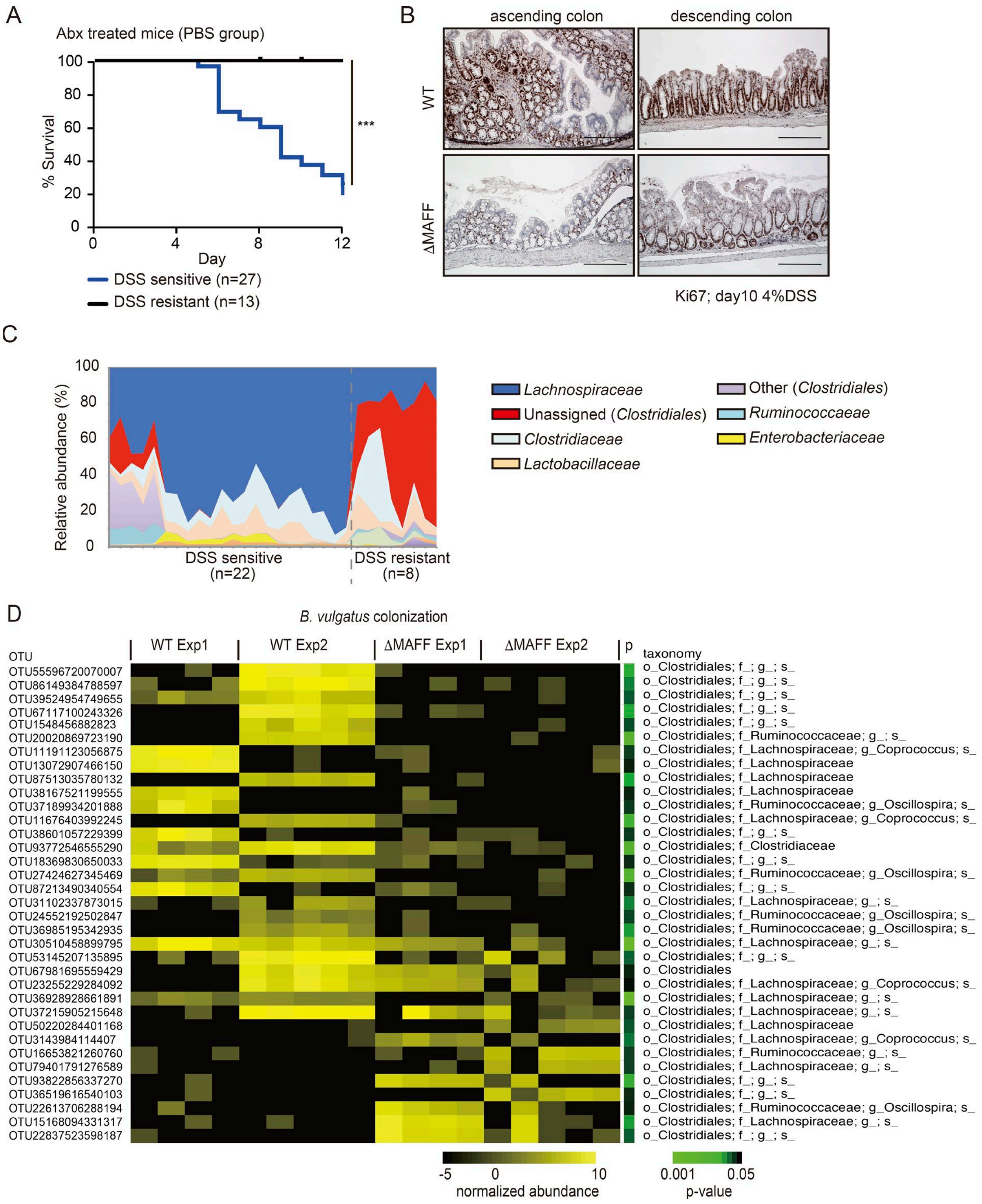

Figure S4. **MAFF system function enhances endogenous colonic members of phylum Firmicutes to induce colonic homeostasis. (A)** C57BL/6 mice were orally inoculated with PBS after the treatment of metronidazole and ciprofloxacin (SPF-Abx mice). The treated mice were kept 4 wk in SPF conditions after stopping the antibiotics, and then 4% DSS was administered in drinking water for 7 d. Kaplan-Meier survival curves of the DSS-sensitive (*n* = 27 mice from five batches of independent antibiotic treatment) and DSS-resistant (n = 13 mice from three batches of independent antibiotic treatment) groups are shown. ***P < 0.001 (log-rank test). **(B)** Immunohistochemical staining of Ki67 in colonic tissue sections of indicated SPF-Abx mice 10 d after initiation of DSS treatment (*n* = 2 independent colons). Scale bars, 200 µm. **(C)** 16S rRNA analysis of the gut microbial composition in fecal samples of the mice shown in A collected immediately before DSS treatment. **(D)** Fecal samples of SPF-Abx mice 4 wk after colonization with WT or ΔMAFF strains of *B. vulgatus* were analyzed for 16S rRNA sequences. The frequencies of operation taxonomic units (OTU) between BVWT- and BVΔMAFF-colonized groups were compared by parametric *t* test, and the OTU with P < 0.05 are shown. Results are pooled from two independent colonization trials. Each column represents an individual animal (*n* = 9 mice for each group).

Tables S1–S7 are provided as an Excel file. Table S1 provides the summary of RNA-Seq reads. Table S2 provides a full set of differentially expressed B. theta genes in cecal contents of backpack animals (BP 7-6 versus BP P3U1). Table S3 gives the full annotation of differentially expressed B. theta genes of BTWT and BTΔMAFF in gnotobiotic mice (Fig. 6 A). Table S4 gives the full annotation of differentially expressed functions of phylum Firmicutes in cecal contents of antibiotics-treated SPF animals colonized with BTWT or BTΔMAFF (Fig. 7 E). Table S5 gives a full set of up-regulated genes in colonic epithelial cells of antibiotics-treated SPF animals colonized with BTWT compared with BTΔMAFF-colonized mice (Fig. 7 C). Table S6 provides the bacterial strains and plasmids used in this study. Table S7 provides sequences of primers used in this study.

References

Edgar, R.C. 2004. MUSCLE: multiple sequence alignment with high accuracy and high throughput. *Nucleic Acids Res.* 32:1792–1797. https://doi.org/10.1093/nar/gkh340

Stamatakis, A. 2014. RAxML version 8: a tool for phylogenetic analysis and post-analysis of large phylogenies. *Bioinformatics.* 30:1312–1313. https://doi.org/10.1093/bioinformatics/btu033
